# Enhancing anti-inflammatory activity of *Eucalyptus camaldulensis* by upregulating secondary metabolites using suspension cultures techniques

**DOI:** 10.1038/s41598-025-34963-8

**Published:** 2026-01-29

**Authors:** Mahrous H. Mahrous, Atef MK Nassar, Fathy K. EL-Fiky, Hala M. Hammoda, Amr El-Hawiet

**Affiliations:** 1 Department of Pharmacognosy, Faculty of Pharmacy, Delta University for Science and Technology, Dakhliya, Egypt; 2 Department of Plant Protection, Faculty of Agriculture, Damanhour University, Damanhour, Egypt; 3 Department of Pharmacognosy, Faculty of Pharmacy, Alexandria University, Alexandria, 21521 Egypt

**Keywords:** *Eucalyptus camaldulensis*, Anti-inflammatory, Antioxidant, Volatile oil, Callus, Plant tissue culture, Liquid chromatography/Mass spectroscopy (LC/MS), Gas chromatography/Mass spectroscopy (GC/MS), Chromatography, Drug discovery and development, Molecular engineering in plants, Environmental biotechnology, Regenerative medicine, Tissue engineering

## Abstract

*Eucalyptus camaldulensis* Dehn (Family *Myrtaceae*) is among the most prominent *Eucalyptus* species, extensively exploited for its anti-inflammatory efficacy. The present study undertakes a comparative phytochemical and pharmacological evaluation of leaf and tissue culture (callus) extracts and their corresponding volatile oils. Methanolic extracts from both plant sources were subjected to liquid chromatography–mass spectrometry (LC/MS), revealing 32 phytoconstituents in leaf extracts and 54 in callus extracts. Identified chemical classes included flavonoids, tannins, coumarins, and phenolic acids, with higher relative abundance in callus-derived extracts. Volatile oil analysis using gas chromatography–mass spectrometry (GC/MS) identified 58 compounds in leaf oil and 52 in callus oil. Quantitative profiling demonstrated a significant elevation in bioactive volatiles within callus oil: 1,8-cineole content showed a 2.1-fold increase compared to leaf oil, and both α-terpineol and sabinene exhibited approximately fourfold increases relative to leaf oil. Bioassays further indicated that callus extracts and volatile oils possess enhanced anti-inflammatory and antioxidant activities, exhibiting higher potency than leaf-derived counterparts. Remarkably, the anti-inflammatory activity of callus volatile oil exceeded that of the standard pharmaceutical agent indomethacin. These findings underscore the potential of in vitro tissue culture as a biotechnological tool for amplifying the yield and bioactivity of secondary metabolites in *E. camaldulensis*.

**Supplementary Information:**

The online version contains supplementary material available at 10.1038/s41598-025-34963-8.

## Introduction

*Eucalyptus camaldulensis* Dehn Family *Myrtaceae* commonly named as long beak eucalyptus is one of the most widely distributed *Eucalyptus* species. Traditionally *E. camaldulensis* is used as an anti-inflammatory, antispasmodic, antipyretic and antimalarial remedy^1^ and for respiratory infectious diseases such as pulmonary TB. Also, it helps decrease a variety of clinical respiratory symptoms, which include nasal sinus infections and coughs^2^. *E. camaldulensis* extract exhibit notable cytotoxic activity against numerous cell lines as Hep-2, MCF-7, HeLa, HepG-2, Caco-2 and HCT-116 in a dose-dependent manner^3^. This is owed to its diverse secondary metabolites’ profile of flavonoids, phenolics, pigments, tannins, terpenes, steroids and saponins^4^. Several studies have underscored the role of phenolic compounds, specifically gallic acid and ellagic acid^5^, which are quantitatively associated with the antioxidant effectiveness, exhibiting a dose-dependent response^6^. In addition to its essential oils that are rich in 1,8-Cinole as a major bioactive compound^2,7,8^.

*E. camaldulensis* antinociceptive action may be attributed to its affinity for different anti-inflammatory receptors such as COX-2, TNF, and IL-1 convertase^9^. Multiple components of *E. camaldulensis* essential oil have been identified to exhibit strong binding affinity to the anti-inflammatory receptors such as cis-sabinol, globulol, α-eudesmol, β-eudesmol, and γ-eudesmol^10^. Remarkably, α-pinene shows maximum suppression of inflammatory action^11^. Furthermore, 1,8-cineol demonstrated a significant reduction in lung inflammation by altering cytokine levels and suppressing key inflammatory pathways^12,13^.

Antioxidant activity in *E. camaldulensis* has been widely investigated due to its significant potential in combating oxidative stress^14^. The *E. camaldulensis* antioxidant capacity is attributed to essential oils with key contributions from monoterpenes such as 1,8-cineole, spathulenol and p-cymene^15,16^. These findings highlight *E. camaldulensis* as a valuable source of natural antioxidants, offering promising applications.

Plant tissue culture technology is a valuable biotechnological tool utilized for various purposes as the production of secondary metabolites, study of functional genes, plants breeding and micropropagation, and crop quality improvement^17^. Plant cell and tissue culture is a potential method for the enhancement of production of phytochemicals from plant material. The concentration of secondary metabolites in plant callus is influenced by the addition of growth regulators (auxin or cytokinin) to the Murashige and Skoog (MS) medium. The concentration of growth regulators is frequently a key factor in the accumulation of secondary metabolites. The growth and secondary metabolites expression in the cultivated plant cells are significantly affected by type and concentration of auxin or cytokinin, as well as the auxin/cytokinin ratio^18^. Despite the vast applications and medicinal uses of *E. camaldulensis*, a relatively limited number of studies for the implementation of plant tissue culture techniques on *E. camaldulensis* have been reported including somatic embryogenesis, genetic transformation, protoplast isolation, and secondary metabolite studies^19^.

For example, Wei et al. showed that the phenolic components of *E. camaldulensis* callus could be increased by physical elicitation as 16 h photoperiod^20^. A mixture of auxins containing indole acetic acid (IAA), indole-3-butyric acid (IBA), Indole-3-Propionic Acid (IPA) and naphthalene acetic acid (NAA) successfully grew roots in 50% of *E. camaldulensis* micro-shoots in a remarkably short period of time^21^. The cotyledonary leaves of *E. camaldulensis* were successful in forming callus on cultivation on MS media. The utilization of Naphthaleneacetic acid (NAA) and 6-Benzylaminopurine (BAP) significantly enhanced the callus formation^22^.While, shoot elongation and rooting at a rate of 80% were effectively achieved through the utilization of activated charcoal^23^.

Remarkably the plant tissue culture techniques were successful in the enhancement of the production of specific volatile oil components from cultivated *E. camaldulensis* plant. Regenerated plants from tissue culture yield greater quantities of 1,8-cineole, *α*-phellandrene, *α*-pinene, and *β*-pinene compared to cultivated plant^24^. Another reported application of *E. camaldulensis* tissue culture is somatic embryogenesis which is affected by type and age of the explant, medium strength and plant growth regulators^25,26^.

In the current work, we are presenting a comprehensive analysis of the chemical composition of the volatile oils and extracts of *E. camaldulensis* leaves and callus that were cultured on MS medium supplemented with 2,4-Dichlorophenoxy acetic acid. In this study, we performed the first comparative analysis and quantification of the chemical composition of volatile oils and extracts from *E. camaldulensis* leaves and callus, using GC/MS and LC/MS techniques. A correlation analysis was conducted to determine the relationship between the chemical profile, and the anti-inflammatory activity which was assessed by measuring the effect on inflammatory mediators such as TNF-α and IL-6. Additionally, the antioxidant activities of the callus extracts were measured using the DPPH assay.

## Experimental

### Plant material

Fresh leaves obtained from plants in their natural habitat. *E. camaldulensis* (dehn) aerial parts were collected in May 2021 during fruiting stage from Antoniades Garden, Smouha Sq., Sidi Gabir, near the Mahmoudia Canal at the southern entrance of Alexandria, Egypt. The exact GPS location co-ordinates are [31.2057° N, 29.9470° E]. All necessary permits, permissions, or licenses for plant material collection were obtained. Voucher specimens (EC052021A) were stored in the herbarium of the Pharmacognosy Department, Faculty of Pharmacy, Delta University for science and technology. Dr. Hesham Mohamed Ali, Professor at Horticulture Research Institute, kindly identified the plants. All experimental research and field studies involving plants (cultivated or wild), including the collection of plant material, were conducted in accordance with relevant institutional, national, and international guidelines and legislation.

#### Preparation of the methanolic extracts of the leaves of E. camaldulensis (dehn) in the specified development stages for UPLC-MS analysis:

Three kilograms of *E. camaldulensis* leaves were collected, shade-dried and ground. Methanol was selected as solvent of interest in the current study due to its broad polarity range and well-established efficiency in extracting both polar and moderately non-polar phytochemicals, particularly phenolic and flavonoid compounds of interest in our biological evaluations^27^. The leaves’ methanolic extract was prepared by collecting the extract after soaking the leaves in methanol for 3–4 days. This process was repeated over a period of one month and the obtained extracts were combined and mixed. TLC screening was performed on the obtained extracts to ensure complete extraction and stability of the secondary metabolites^28^. To minimize the impact of co-extracted pigments or waxes, the extracts were subjected to filtration and centrifugation steps, and in some cases stored at low temperatures to allow sedimentation of insoluble residues. The methanolic extracts were concentrated under reduced pressure and the dry residues were used to prepare extracts of concentration 1 mg/ml dissolved in HPLC-grade methanol followed by their filtration via membrane Disc Filter PTFE, (0.2 μm). The prepared extracts were sonicated before introduction and injection on the chromatographic separation column that receives a volume of 10 µl of each sample in the full loop mode. Each sample is analyzed in triplicate manner.

#### Preparation of essential oils

The essential oils of the plant species studied were prepared by hydro-distillation for 2 Kg. of fresh leaves using Clevenger type apparatus for 4 h. Each oil extraction was carried out in triplicates. The oils were desiccated over anhydrous Na_2_SO_4_ followed by oils storage in sealed brown vials at −18 °C until further spectral analyses and biological evaluation.

### Initiation of static and suspension cultures of *E. camaldulensis*

#### Total methanolic extract of callus

Identically sized cut leaves of *E. camaldulensis* were thoroughly washed with abundant tap water to completely remove any dust. The surfaces of the cut leaves were then sterilized by immersion in 70% ethanol for 5 min, followed by 20 min in 20% (v/v) sodium hypochlorite. The forceps were flame sterilized by soaking them in ethanol and inserting them into the spirit lamp. Furtherly, the cut leaves were properly washed with sterile distilled water 3 times before being blotted dry on sterile filter paper. The sterile leaf segments were placed on a culture medium containing sucrose (30 g/L), agar (14 g/L), and 2,4-D growth regulator (4 mg/L). The 2,4-D concentration of 4 mg/L has been chosen after a pre-optimization experiment by using increasing concentrations of 2,4-D from 1 mg/L till 6 mg/L where concentration of 4 mg/L showed the maximum callus production^29,30^. The media was pH-adjusted to a range of 5.8–6.2 before being subjected to autoclaving at a temperature of 121 °C and a pressure of 15 psi for a duration of 20 min^31^. After four weeks of incubation, only well-developed regenerating callus with a yellow-white or green friable appearance, characterized by a soft, crumbly mass of plant cells, were selected. This callus was in the form of (3 mm) pieces and were obtained from the optimal concentration of 2,4-D (4 mg/L). Suspension cultures were initiated by subculturing 2–3 g callus onto 100 ml liquid medium. Long-necked Erlenmeyer flasks were used and placed on an orbital shaker incubator at 120 rpm. Small clumps of cells were formed after 2 weeks. Subculturing was performed at 1- to 3-week intervals, depending on the number of clamps produced. After incubation and subculturing of the suspension culture on an orbital shaker (120 rpm), at 23–25℃ for 16 weeks. Extended 16-week period was designed to promote the gradual enlargement of callus mass and enhance the accumulation of secondary metabolites. To maintain healthy growth conditions and avoid nutrient depletion, we performed regular subculturing every two weeks. This strategy allowed us to sustain cell viability and vigor over the longer cultivation period. Callus of liquid medium was weighed after filtration. A total of 45.5 g of fresh proliferated callus tissue was harvested after the 16-week culture period. The biomass was subjected to methanol extraction, and the resulting extract was concentrated under reduced pressure. The final yield of the dried extract was 1.2 g, corresponding to a percentage yield of 2.64% based on the initial fresh weight of the callus. To ensure sterile conditions were maintained prior to the experimental procedures, we prepared and incubated uninoculated plates under standard conditions for a period of two weeks. These plates remained free of microbial growth, confirming the effectiveness of our sterilization protocol. Furthermore, during the cultivation phase, an empty control plate was maintained alongside the cultured samples to continuously monitor for potential contamination. No signs of microbial growth were observed in any of the control plates, supporting the reliability and consistency of the sterile conditions throughout the experimental process.

#### Callus volatile oil separation

The sterile leaf segments were placed on a culture medium containing sucrose (30 g/L) and agar (14 g/L). Cell suspensions were created by transferring callus from static cultures into the suitable MS liquid media, which was enriched with 30 g/L sucrose and 4 mg/L of the growth regulator 2,4-D. The pH of the media was adjusted to a range of 5.8–6.2 before subjecting it to autoclaving at a temperature of 121 °C and a pressure of 15 psi for a duration of 20 min. The flasks were placed on an orbital shaker, which was set to rotate at a speed of 120 rpm. The temperature within the shaker was maintained between 23 and 25 °C. This incubation process lasted for a period of 8 weeks. The liquid media was combined within a 1 L clean sterile flask. One cell-disruption technique aimed at enhancing the extraction efficiency of intracellular constituents from callus tissue. Specifically, after decanting the cell masses from the suspension culture, the harvested callus was triturated in a sterile mortar using pre-washed, heat-sterilized sea sand. This step served to mechanically abrade and rupture the callus surface, effectively exposing internal cell contents and increasing the contact area for subsequent methanol extraction. Following trituration, the entire mixture of disrupted cell mass and the residual culture liquid was combined to ensure no loss of soluble metabolites, then filtered through Whatman Grade 1 filter paper. This method was developed as a simple, low-cost alternative to enzymatic or mechanical homogenization, and it contributed to a notably higher extract yield compared to conventional extraction from intact callus^32^. Solid sample is mixed with all liquid present in suspension cultures then filtered using Whatman Grade 1. Separated liquid is kept at 4 °C for 2 days. Using benchtop centrifuge, liquid form 2 layers oil and aqueous. Volatile oil is collected using a separating funnel.

### Identification of compounds by LC/MS and GC-MS analysis

#### Samples and standards Preparation for UPLC-MS analysis

The preparation of the samples and standards was carried out in accordance to reported protocols^33^. 0.22 μm membrane disc filter was used to filter each dried plant extract at a concentration of 10 mg/mL after being diluted in HPLC-grade methanol. Moreover, samples were sonicated to eliminate gas prior to injection. The chromatographic column was loaded with samples using injection volumes of 10 µL and full loop mode injection. The sample analysis was performed 3 times. Besides, quality control (QC) samples were created by combining 5 µL aliquots of each sample for the purpose of standardization of the data and assessing the stability and repeatability of the proposed analytical platform.

#### UPLC and ESI-MS conditions

Ultra-performance liquid chromatography system with a mass spectrometer, manufactured by Waters Corporation, Milford, USA. The reverse-phase separations were conducted using an ACQUITY UPLC BEH C18 Column with dimensions of 2.1 × 50 mm and an inner diameter of 1.2 mm. The particle size of the column was 1.7 μm. The separations were performed at a flow rate of 0.2 m/mL. The analyses were conducted using a mobile phase composed of water acidified with 0.1% formic acid (A) and methanol acidified with 0.1% formic acid (B). The elution conditions used were as follows: from 0 to 2 min, isocratic elution at 10% B; from 2 to 5 min, a linear increase from 10% to 30% B; from 5 to 15 min, a linear increase from 30% to 70% B; from 15 to 22 min, a linear increase from 70% to 90% B; and from 22 to 25 min, isocratic elution at 90% B. Finally, the column was washed and reconditioned^34^.

#### Conditions for ESI-MS and annotation of metabolites

The analysis was carried out using electrospray ionization quadrupole linear ion trap-tandem mass spectrometry, with both positive and negative ion acquisition modes. The instrument used for the analysis was UHPLC-XEVO- QqQ- MS/MS analysis ultraperformance liquid chromatography coupled to triple quadruple instrument Waters Corporation, Milford, MA01757 U.S.A. Electrospray ionization (ESI) was conducted in both negative and positive ionization modes. The analysis parameters were configured in negative ion mode with the following settings: cone voltage of 30 electron volts, capillary voltage of 3 kilovolts, cone gas flow rate of 50 L per hour, source temperature of 150 °C, desolvation temperature of 440 °C, and desolvation gas flow rate of 900 L/hr. Mass spectra were seen in the electrospray ionization (ESI) method within the range of m/z 100 to 1000 atomic mass units. The chemical components were identified based on their ESI–QqQLIT–MS/MS spectra and fragmentation patterns. The parent ion is selected in the first quadrupole (Q1) and fragmented in the second quadrupole (collision cell) using the collision-induced dissociation (CID) method with a 30 to 70 eV energy ramp and nitrogen gas as a collision gas. Molecular weight (MW), retention time (tR), fragmentation pattern, and a specific ultraviolet (UV) spectrum were used to simultaneously and directly match the discovered molecules with pure standard references in the in-house library for identification of compounds. The semi-quantitative analysis of all the metabolites in the extracts were quantified as milligram standard equivalents per gram dry weight using calibration curves. Stock solutions (10 mg) of Kaempferol-4′- glucoside, Caffeic acid, Gallic acid, and Maslinic acid were prepared in UPLC-grade methanol within 10 mL volumetric flasks. These were serially diluted with methanol to working concentrations (0.01–0.70 mg/mL). Each concentration level (5µL injection) was analyzed in duplicate by UPLC. Calibration curves were generated by plotting the resulting peak areas against the known standard concentrations. The developed quantitation method was then evaluated and validated following ICH (The International Council for Harmonisation of Technical Requirements for Pharmaceuticals for Human Use) guidelines in terms of linearity, sensitivity, limit of detection (LOD) and limit of quantitation (LOQ) (Details in Table S.1)^35^.

The identification of the metabolites was performed by comparing their retention times to that of external standards. Based on in-house database and phytochemical dictionary of natural products database (CRC), the annotation of the metabolites was performed using quasi-molecular ions and characteristic MS/MS fragments. The annotation, according to the Metabolomics Standards Initiative (MSI), was either confidence level 1 (using genuine standards investigated under the same experimental parameters) or confidence level 2 (using the same experimental settings)^36^.

#### GC-MS analysis of *E. camaldulensis* volatile oil

An aliquot of approximately 1 µL was extracted and subjected to analysis using Gas Chromatography-Mass Spectrometry (GCMS) in conjunction with the Varian 8200 autoinjector. The semi-quantitative analysis utilizing GC-MS was conducted using the Agilent 7890 A Gas Chromatography equipment paired with an Agilent 5975B mass selective detector. The GC-MS system was fitted with an HP-5 capillary column (30 m ×0.25 mm ID, film thickness 0.25 μm, Agilent Technologies, USA).

The separation of chemical components was achieved under the specified conditions: the starting column temperature was set at 50 °C for a duration of 6 min. The temperature was raised to 215 °C at a rate of 15 °C per minute and held for 1 min. It was then further increased to 230 °C at a rate of 5 °C per minute and eventually raised to 290 °C at the same rate. The temperature was held at 290 °C for 2 min. The solvent delay was maintained at 4 min, with the injector temperature set to 250 °C and helium gas employed as the carrier. The electron ionization mass spectra were measured up to 800 m/z using an ion source temperature of 230 °C and an energy of 70 eV. The GC-MS instrument was operated using a computer system equipped with EI-MS libraries. The chemicals were identified through the analysis of their entire mass spectra scans and retention time, utilizing their total ion chromatograms (TIC). The full scan mode enabled the comparison of the compound spectrum with the EI-MS database libraries, such as NIST14 and Willey9, to determine the contrast.

### Biological activities

#### Assessment of the anti-inflammatory activity of *E. camaldulensis* leaf and callus extracts

The WI-38 Cells were cultured in a 5% CO_2_ environment at a temperature of 37 °C for a duration of 24 h until 90% of the cells had reached confluence. WI-38 cells were pre-treated with the tested extracts at serial concentrations of (25, 50, 100–200 µg/mL) or vehicle control for 2 h prior to lipopolysaccharides (LPS) stimulation. This pre-incubation period is a standard practice in similar anti-inflammatory assays to allow sufficient cellular uptake and interaction of bioactive constituents with intracellular targets^37^. Then, Lipopolysaccharides were added at a concentration of 0.5 µg/mL, and the incubation continued with 10% fetal bovine serum, 100 U/mL penicillin, and 100 µg/mL streptomycin at a temperature of 37 °C with 5% CO_2_ for a duration of 22 h. Following the treatment, the culture media and cells were collected individually. Cell viability was assessed using the MTT assay at the end of the 22-hour treatment period. The results confirmed that cell viability remained above 90% across all treated groups, including those exposed to extracts and LPS, indicating that the tested concentrations were non-cytotoxic under the experimental conditions. The collected media were subjected to centrifugation at a speed of 1000 rpm for a duration of 10 min. The levels of TNF-α and IL-6 in the media were measured using mouse TNF-α and mouse IL-6 ELISA kits, following the instructions provided by the manufacturer. The absorbance at 450 nm was used to determine the levels. The overall concentration of the inflammatory factor in the media was adjusted to account for the total protein content of the viable cell pellets. The samples exhibit color variations as a manifestation of the obtained outcomes. The addition of antibody reagents resulted in the appearance of a blue tint, which then changed to yellow upon the addition of the reaction terminator. The extracts were assessed for their anti-inflammatory properties against LPS-induced inflammation in WI-38 cell lines by quantifying the expression of TNF-α and IL-6 using ELISA.

The choice of WI-38 cell line was based on its remarkably stable genetic signatures. Chen, A., et al., 2025 demonstrated the absence of significant Short Tandem Repeat (STR) drift for WI-38 supporting its continued use without frequent reauthentication despite prolonged propagation and storage. This genomic consistency affirms the authenticity of WI-38 and minimizes concerns regarding contamination or phenotypic variability commonly observed in long-term cell cultures^38^. All cell lines in this work were gifted by Dr. Farid Badria, Professor at Faculty of Pharmacy, Mansoura university.

WI-38 cells were placed in a 96-well plate at a concentration of 1 × 10^5^ cells per milliliter. The following day, the cells were subjected to treatment with extracts that were determined to be non-toxic to the cells, followed by treatment with LPS at a concentration of 0.5 µg/mL. Alternatively, the cells were treated with LPS. Following the incubation period, the liquid containing the culture was removed by suction and then subjected to centrifugal force at a speed of 1000 rpm for a duration of 10 min. The liquid portion of the mixture was gathered and the levels of TNF-α and IL-6 were assessed in each individual sample. The levels of TNF-α and IL-6 in the media were quantified using an ELISA and standardized based on the protein concentration.

The anti-inflammatory effect of the tested samples was evaluated by measuring the levels of TNF-α and IL-6 released into the culture supernatant of LPS-stimulated cells. For each experimental group (normal control, LPS control, and Drug + LPS treatments), cytokine concentrations (pg/mL) were determined in triplicate, and the mean values were used for further analysis. To normalize the data to the inflammatory stimulus, the percentage inhibition of cytokine production was calculated relative to the LPS control group, which was considered as 0% inhibition. For each concentration of the samples tested, the percentage inhibition vs. LPS was calculated according to the following equation:

1 $$\:\mathrm{\%}\mathrm{\:inhibition\:vs\:LPS}=(1-\frac{{Cytokine}_{\mathrm{Drug+LPS}}}{{Cytokine}_{\mathrm{LPS\:only}}})\times\:100$$

where *Cytokine*₍Drug+LPS₎ is the mean TNF-α or IL-6 level in the presence of the tested sample plus LPS, and *Cytokine*₍LPS only₎ is the mean level in cells treated with LPS alone. The same formula was applied separately for TNF-α and IL-6. The concentration–response relationship and the half-maximal inhibitory concentration (IC₅₀) values of each sample were obtained using non-linear regression in GraphPad Prism 10 (GraphPad Software, Inc., San Diego, CA, USA). For each sample and each cytokine, an XY data table was created with the sample concentration (µg/mL) as the X variable and the corresponding percentage inhibition vs. LPS as the Y variable. The data were fitted using the log(inhibitor) vs. response model (dose–response, inhibition).

The following four-parameter logistic equation was used:

2 $$\:Y=\mathrm{Bottom}+\frac{\mathrm{Top}-\mathrm{Bottom}}{1+{10}^{(\mathrm{l}\mathrm{o}\mathrm{g}I{C}_{50}-X)\times\:\mathrm{HillSlope}}}$$

where *Y* is the percentage inhibition, *X* is the logarithm of the sample concentration, *Top* and *Bottom* represent the upper and lower plateaus of the curve, *HillSlope* describes the steepness of the curve, and IC₅₀ is the concentration producing 50% inhibition. Parameters were estimated by non-linear least squares, and IC₅₀ values with their 95% confidence intervals were obtained from the fitted curves. The non-steroidal anti-inflammatory drug Piroxicam (10 µg/mL) was included as a reference standard for comparison.

#### Assessment of the antioxidant activity of *E. camaldulensis* leaf and callus extracts

The antioxidant ability of the examined extracts was evaluated using the DPPH (1,1-diphenyl-2-picryl-hydrazyl) assay, which measures redox potential through the reduction of a stable free radical that appears deep violet due to its broad electron delocalization and strong absorption at 515–517 nm when dissolved in methanol. A 1 mM DPPH stock solution (0.394 mg/mL) was prepared and diluted 1:10 to obtain a 100 µM working solution, which was further adjusted to yield an initial optical density (OD) of approximately 0.7 at 517 nm. This OD value falls within the optimal range (0.6–0.8) for ensuring sensitivity, linearity, and sufficient dynamic range in detecting radical scavenging activity, while minimizing background interference or signal saturation^39^. The absorbance of this solution at 517 nm was measured to be between 0.6 and 0.7.

A total of 500 µl of the sample and 500 µl of a 100 µM DPPH solution were combined in a cuvette. Additionally, negative control was made by mixing 500 µl of methanol with 500 µl of DPPH solution. Both solutions were placed in a dark environment at room temperature for a duration of 15 min. The absorbance was measured at a wavelength of 517 nm using methanol as the reference solution. Results were expressed as the percentage of reduction of the radical absorbance^40^.

% inhibition = ([(Abs max (negative control)) – (Abs sample + DPPH)]/Abs max) X 100.

The extract’s antioxidant activity was measured by comparing it to Trolox, a known antioxidant agent. The results are presented as Trolox equivalent antioxidant capacity (TEAC). A calibration curve was created to establish the relationship between the percentage of inhibition and the concentrations of Trolox, ranging from 12.5 to 0.3 µg/ml.

Calibration curve between inhibition percentage at various concentrations of Trolox when it mixed with DPPH and concentration of Trolox shows strong linearity was demonstrated by the high correlation coefficient value of 0.9915. The TEAC (Trolox Equivalent Antioxidant Capacity) of the extract was calculated by substituting in the inhibition percentage of the tested extract into the regression equation of the calibration curve (S.4).

### Statistical analysis

All statistical analyses were performed using GraphPad Prism 10 (GraphPad Software, Inc., San Diego, CA, USA). Data are presented as mean ± standard deviation unless otherwise stated. Pearson correlation coefficients (r) were calculated to assess the relationships between TNF-α and IL-6 inhibition, as well as between chemical composition (e.g., 1,8-cineole content) and cytokine inhibition. A p-value < 0.05 was considered statistically significant.

## Results and discussion

### Callus induction

*E. camaldulensis* leaves’ explants were cultured and callus were produced directly using MS media supplemented with (2,4-D,4) growth hormone. After 4 weeks, all the leaf explants were converted into a yellowish white callus fragile mass. After 2 weeks all the leaf explants were converted into a yellowish-brown callus. The brownish coloration of the formed callus and even leaves themselves in the static media is due to partial oxidation of phenolic content. The formed callus were transferred into a liquid media containing (2, 4- D, 4) as growth regulators, subcultured on a biweekly basis. The developed callus were extracted a minimum of three times using methanol. as mentioned in Sect. 2.2.

### Comparison of *E. camaldulensis* leaf, and its callus extracts using UPLC/MS analysis

**Fig. 1:**
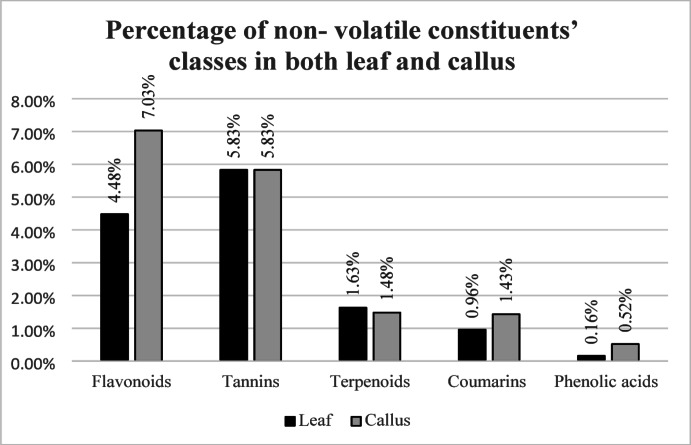
Comparison between percentage of constituents’ classes in both leaf and callus.

The UPLC-MS analysis of the examined extracts showed a wide, qualitative and quantitative, range of phytochemical constituents. The chief ingredients detected in *E. camaldulensis* leaf and induced callus were tannins, flavonoids and terpenoids. Thirty-two compounds in *E. camaldulensis* leaf extract were detected as tannins, flavonoids, terpenoids, coumarins and phenolic acids that represent 5.83%, 4.48%, 1.63%, 0.96% and 0.16% of total methanolic extract, respectively. whereas fifty-four constituents were detected in *E. camaldulensis* callus mainly as flavonoids, tannins, terpenoids, coumarins and phenolic acids representing 7.03%, 5.83%, 1.48%, 1.43% and 0.52% of total methanolic extract, respectively. Comparison between percentage of constituents’ classes in both leaf and callus is shown in Fig. 1.

A total of 52 compounds were identified in the methanolic extract of *E. camaldulensis* leaf (Figure S.1) and methanolic extract of *E. camaldulensis* callus chromatograms (Figure S.2), with the most prevalent classes being tannin, flavonoid, phenolic acid, fatty acid, terpenoid, coumarin, glycoside, steroid and fatty alcohol. The identified secondary metabolites of *E. camaldulensis* leaf methanolic extract and callus methanolic extract are listed in Tables 1 and 2.

**Table 1:** Metabolites identified in *Eucalyptus camaldulensis* leaves sample extract using UPLC-MS in negative ionization mode:.

#	tR	Identified compounds	Precursor ion	Molecular weight	MS/MS	Reference	Elemental composition	Class
1	0.77	4-glucogallic acid	331.0895 (M-H)	332.26	169, 151, 125	^41^	C13H16O10	Tannin
2	0.77	Caffeic acid *	215.039 (M+Cl)	180.16	179, 161, 135, 109, 91	^42^	C9H8O4	Phenolic acid
3	0.77	Epicatechin hexoside	487.186 (M+Cl)	452.4	451.1, 289.1	^43^	C21H24O11	Tannin
4	0.77	Gallic acid gallate	160.7793 [(M/2)-H]	322.22	321.02, 169.01, 125.02, 124.01	^44^	C14H10O9	Tannin
5	0.77	galloyl quinic acid	379.074 (M+Cl)	344.27	343.0687, 191, 169, 125	^45^	C14H16O10	Tannin
6	0.77	Malic acid	168.9921 (M+Cl)	134.09	133, 115	^42^	C4H6O5	acid
7	1.05	Gallic acid *	168.9917 (M-H)	170.12	169.02, 151.02, 125.02, 124.01, 123.01, 107.01	^44^	C7H6O5	Tannin
8	12.85	(S)-rutaretin	297.0829 (M+Cl)	262.26	261.07, 246.05, 231.02, 218.05, 203.03	^46^	C14H14O5	Coumarin
9	13.6	(Epi) catechin	311.1293 [(M-2 H)+Na]	290.27	289.0722, 245, 205, 203	^45^	C15H14O6	Tannin
10	13.78	Aspidinol	268.1812 (M+HCOO-H)	224.25	223, 207, 179, 151	^47^	C12H16O4	Phenolic acid
11	13.78	Dalpaniculin	267.0935 [(M/2)-H]	536.5	535.1457	^8^	C25H28O13	Flavonoid
12	14.31	Macrocarpal I	489.3591 (M-H)	490.6	489.2852, 489, 207	^48^	C28H42O7	Terpenoid
13	14.31	Quercetin-hexoside	499.2346 (M+Cl)	464.4	463.0860, 301, 300, 343	^49^	C21H20O12	Flavonoid
14	14.58	Maslinic acid *	471.4041 (M-H)	472.7	423.33, 405.32, 393.31, 377.29	^8^	C30H48O4	Terpenoid
15	14.87	Galloylchlorogenic acid	252.1672 [(M/2)-H]	506.4	505, 343	^50^	C23H22O13	Tannin
16	16.14	Vitexin (apigenin 8-C glucoside)	431.1617 (M-H)	432.4	431, 341, 311, 269	^51^	C21H20O10	Flavonoid
17	16.63	3-tert-butyl-5-methylcatechol	404.3177 (2 M+HCOO-H)	180.24	180.247 [M + H]+, 93.07, 149.06, 121.065	^52^	C11H16O2	Tannin
18	17.24	Kaempferol-4′-hexoside *	485.3461 [(M-2 H)+K]	448.4	447.0925, 352.0669, 285.0406	^53^	C21H20O11	Flavonoid
19	17.45	Ellagic acid hexoside	485.2988 [(M-2 H)+Na]	464.3	132, 145, 172, 216, 244, 284, 301	^54^	C20H16O13	Tannin
20	17.61	Eucalypcamal A	417.174 (M-H)	418.2	419.2067 [M + H]+	^55^	C23H30O7	Terpenoid
21	17.61	Quercetin rhamnoside (astragalin)	485.3229 (M-2 H)+K	448.4	447.0927, 284, 285, 327	^49^	C21H20O11	Flavonoid
22	17.96	Eucalypcamal N	417.1755 (M-H)	418.487	417.1921	^8^	C23H30O7	Terpenoid
23	17.96	Rumexoside	457.3017 (M+Cl)	422.4	421.1138	^8^	C20H22O10	Glycoside
24	18.5	Epigallocatechin gallate	493.1311 (M+Cl)	458.4	457, 331, 305, 169	^56^	C22H18O11	Tannin
25	18.5	Methyl gallate	221.1105 (M-2 H)+K	184.15	168.0, 140.0, 124.0, 111.1	^57^	C8H8O5	Tannin
26	18.5	Αlpha-peroxyachifolide	375.2261 (M-H)	376.1523	375.145	^8,58^	C20H24O7	Terpenoid
27	18.58	Epicatechin gallate	486.2536 (M+HCOO-H)	442.4	441, 331, 289, 169	^59^	C22H18O10	Tannin
28	18.58	Hydrojuglone glucoside	375.2128 [(M-2 H)+K]	338.31	337, 175, 157, 147, 131	^60^	C16H18O8	Glycoside
29	18.58	valoneic acid dilactone	507.1812 [(M-2 H)+K]	470.3	469, 425, 301	^61^	C21H10O13	Phenolic acid
30	18.7	Luteolin-6-c-β-d- pentose	455.3931 [(M-2 H)+K]	418.3	417.0826	^8^	C20H18O10	Flavonoid
31	27.04	Eucalypcamal M	401.1371 (M-H)	402.488	401.1967	^8^	C23H30O6	Terpenoid
32	27.04	Phloretin	547.929 (2 M-H)	274.27	273, 167	^62^	C15H14O5	Phenolic acid

* Compound identified using reference standard.

**Table 2:** Metabolites identified in *Eucalyptus camaldulensis* callus sample extract using UPLC-MS in negative ionization mode:.

#	tR	Identified compounds	Precursor ion	Molecular weight	MS/MS	Reference	Elemental composition	Class
1	0.77	4-glucogallic acid	331.09 (M-H)	332.26	169, 151, 125	^41^	C13H16O10	Tannin
2	0.77	Caffeic acid *	217.04 (M-2 H+K)	180.16	179, 161, 135, 109, 91	^42^	C9H8O4	Phenolic acid
3	0.77	Docosanoic acid	377.11 (M-2 H+K)	340.6	339, 321, 295, 59	^63^	C22H44O2	Fatty acid
4	0.77	Galloyl glucose	331.09 (M-H)	332.26	169, 271, 313, 331	^64^	C13H16O10	Tannin
5	0.77	galloyl quinic acid	379.11 (M+Cl)	344.27	343.0687, 191, 169, 125	^45^	C18H24O10	Tannin
6	0.77	galloylglucopyranose	331.09 (M-H)	332.26	169, 271, 313, 331	^64^	C13H16O10	Tannin
7	0.77	Phloretic acid	331.09 (2 M-H)	166.17	165.0553, 121.0659	^65^	C9H10O3	Phenolic acid
8	1.05	Coumaroylquinic acid	168.99 (M/2-H)	338.31	337, 279, 191, 173, 163	^66^	C16H18O8	Coumarin
9	1.05	Hexahydroxydiphenic acid	168.99 (M/2-H)	338.22	339.2, 322, 182, 109	^67^	C14H10O10	Phenolic acid
10	7.8	Dalpaniculin	557.35 (M-2 H+Na)	536.5	535.1457	^8^	C25H28O13	Flavonoid
11	8.93	Galloylshikimic acid	696.5 (2 M+HCOO-H)	326.25	325.0577, 281, 173, 169, 125	^45^	C14H14O9	Phenolic acid
12	9.22	Caffeoylquinic acid	398.22 (M+HCOO-H)	354.31	353, 191, 179, 173	^68^	C16H18O9	Phenolic acid
13	9.22	Chlorogenic acid	398.22 (M+HCOO-H)	354.31	353, 191, 179, 173, 135	^69^	C16H18O9	Phenolic acid
14	9.22	Tricosanoic acid	398.22 (M+HCOO-H)	354.6	353, 181	^70^	C23H46O2	Fatty acid
15	10.54	Isosyringinoside	266.17 (M/2-H)	534.5	[M + NH4]+ 552.22, 193.08, 161.05	^71^	C23H34O14	Flavonoid
16	12.87	(S)-rutaretin	297.1 (M+Cl)	262.26	261.08, 246.06, 231.03, 218.06, 203.04	^46^	C14H14O5	Coumarin
17	13.62	Eucalypcamal A	453.36 (M+Cl)	418.487	419.2067 [M + H]+	^55^	C23H30O7	Terpenoid
18	13.62	Eucalypcamal N	453.36 (M+Cl)	418.487	417.1921	^8^	C23H30O7	Terpenoid
19	13.62	Phloretin	311.05 (M-2 H+K)	274.27	273, 167	^62^	C15H14O5	Phenolic acid
20	13.82	Quercetin pentoside (Quercetin 3-O-Alpha-L-Arabinoside)	471.41 (M-2 H+K)	434.3	433, 301, 291, 285, 247	^72^	C20H18O11	Flavonoid
21	13.82	Tricetin-3′-xyloside	471.41 (M-2 H+K)	434.3	300.0, 282.8, 232.9, 228.9.	^73^	C20H18O11	Flavonoid
22	16.05	Ellagic acid hexoside	485.26 (M-2 H+Na)	464.3	132, 145, 172, 216, 244, 284, 301	^54^	C20H16O13	Tannin
23	16.05	Isovitexin	431.14 (M-H)	432.4	431.1432, 341, 311, 283, 117	^74^	C21H20O10	Flavonoid
24	16.05	Procyanidin trimer	432.15 (M/2-H)	866.8	865, 577, 289	^75^	C45H38O18	Flavonoid
25	16.05	Quercetin-hexoside	485.26 (M-2 H+Na)	464.4	463.0860, 301, 300, 343	^49^	C21H20O12	Flavonoid
26	16.05	Vitexin (apigenin 8-C glucoside)	431.14 (M-H)	432.4	431, 341, 311, 269	^51^	C21H20O10	Flavonoid
27	16.53	(Epi) catechin	325.23 (M+Cl)	290.27	289.0722, 245, 205, 203	^45^	C15H14O6	Tannin
28	16.53	Catechin	325.23 (M+Cl)	290.27	289.0722, 245, 205, 179	^76^	C15H14O6	Tannin
29	16.53	cis-9-Octadecenoic acid	326.28 (M+HCOO-H)	282.5	117, 341	^77^	C18H34O2	Fatty acid
30	16.64	3-tert-butyl-5-methylcatechol	404.27 (2 M+HCOO-H)	180.24	180.247 [M + H]+, 93.07, 149.06.	^52^	C11H16O2	Tannin
31	16.64	Tetracosanoic acid	404.27 (M+Cl)	368.6	117, 425	^77^	C24H48O2	Fatty acid
32	17.29	Epicatechin gallate	486.34 (M+HCOO-H)	442.4	441, 331, 289, 169	^59^	C22H18O10	Tannin
33	17.5	Kaempferol-4′-glucoside *	485.3 (M-2 H+K)	448.4	447.0925, 352.0669, 285.0406	^53^	C21H20O11	Flavonoid
34	18	Cnidilin	337.26 (M-2 H+K)	300.3	299.1, 233.05, 218.03, 173.02	^78^	C17H16O5	Coumarin
35	18	Ellagic acid	337.26 (M+Cl)	302.19	301, 284, 255.2, 184.7	^67^	C14H6O8	Tannin
36	18	Hesperetin	337.26 (M+Cl)	302.28	301.0711, 285, 267, 241, 173	^79^	C16H14O6	Flavonoid
37	18	Hydrojuglone glucoside	337.26 (M-H)	338.31	337, 175, 157, 147, 131	^60^	C16H18O8	Glycoside
38	18	Protocatechuic acid-hexoside	337.26 (M-2 H+Na)	316	315, 153	^80^	C13H16O9	Glycoside
39	18	Quercetin	337.26 (M+Cl)	302.23	301, 257.4, 229.2, 185.1	^67^	C15H10O7	Flavonoid
40	18	Quercetin-rutinoside (rutin)	647.51 (M-2 H+K)	610.5	610.6, 464, 301, 283.4	^67^	C27H30O16	Flavonoid
41	18	Tricetin	337.26 (M+Cl)	302.23	301.19, 273.13, 229.1, 179.07.	^81^	C15H10O7	Flavonoid
42	18.77	Stigmast-4-en-3-one	456.41 (M+HCOO-H)	412.7	412, 397, 370, 288, 271, 229.	^82^	C29H48O	Steroid
43	18.95	Epicatechin glucoside	487.37 (M+Cl)	452.4	451.1, 289.1	^43^	C21H24O11	Tannin
44	21.15	Casuarinin	467.3 (M/2-H)	936.6	935.50, 633, 615, 659, 571, 481	^83^	C41H28O26	Tannin
45	21.42	Maslinic acid *	471.31 (M-H)	472.7	423.33, 405.32, 393.31, 377.29	^8^	C30H48O4	Triterpene
46	25.54	benzyl-galloylglucose	443.04 (M-2 H+Na)	422.11	421, 331, 313, 169, 151, 125	^84^	C20H22O10	Tannin
47	25.54	Epicatechin	144.85 (M/2-H)	290.27	289.0722, 245, 205, 203	^45^	C15H14O6	Tannin
48	25.54	Epigallocatechin-3-O- p- coumarate	451.17 (M-H)	452.4	451.1035	^85^	C24H20O9	Tannin
49	25.54	Luteolin-7-O-β-D-glucoside	485.34 (M-2 H+K)	448.4	285, 284, 327, 257	^49^	C21H20O11	Flavonoid
50	25.54	Tetracosan-1-ol	708.42 (2 M-H)	354.7	411, 354, 103	^77^	C24H50O	Fatty Alcohol
51	26.38	Beta-Sitostanol	453.31 (M-2 H+K)	416.7	417, 395 [M+H−H2O]+	^86^	C29H52O	Steroid
52	26.77	Castalagin	971.39 (M-2 H+K)	934.6	933, 466, 301	^67^	C41H26O26	Tannin
53	31.07	Icosanoic acid	311.2 (M-H)	312.5	311.2	^87^	C20H40O2	Fatty acid
54	31.07	valoneic acid dilactone	469.29 (M-H)	470.3	469, 425, 301	^61^	C21H10O13	Phenolic acid

* Compound identified using reference standard.

Identification of all the detected compounds was performed as previously reported through comparing the detected compounds with standard references based on their molecular weight (MW), retention time (tR) and fragmentation pattern^36^. Despite the fact that the quantities of detected phytochemical classes were similar in both callus and leaf methanolic extracts, however the quantity of flavonoids was doubled in callus extract. Similarly, coumarins were detected as 1.5 folds in callus extract compared to leaf extract.

Individual tracking of changes in members of each detected phytochemical class showed notable differences in the phytochemical pattern between leaf and callus extracts. Several flavonoids were uniquely detected in callus extract as hesperetin, isosyringinoside, isovitexin, procyanidin trimer, quercetin, quercetin-rutinoside (rutin), tricetin, luteolin − 7-O-β-D-glucoside and tricetin-3′-xyloside. While callus extract was found to be rich in tannin derivatives as galloyl glucose, catechin, ellagic acid, casuarinin, benzyl-galloylglucose, epicatechin, epigallocatechin-3-o-p-coumarate and castalagin which were not detected in leaf extracts. Remarkably, 4-glucogallic acid was detected in leaves and in callus, while galloyl glucose was expressed only in callus.

Regarding phenolic acids, phloretin was detected in both leaf and callus extracts while phloretic acid, hexahydroxydiphenic acid, galloylshikimic acid and caffeoylquinic acid (chlorogenic acid) were exclusively detected in callus extracts. Similarly, callus extract was remarkably enriched with specific terpenoids and coumrin compounds that are not detected in leaf extracts as macrocarpal I, α-peroxyachifolide, eucalypcamal M, coumaroylquinic acid and cnidilin appeared in callus chromatogram only.

### Comparison of *E. camaldulensis* leaf, and its callus volatile oils using GC/MS analysis

*E. camaldulensis* essential oil was extracted from both leaf and suspension culture to measure the percentage yield of each volatile extract. Oil produced by callus (VOC) (2.07 ± 0.12%% v/w) with fifty-two constituents was found to be more than that produced from leaf (VOL) (1.74 ± 0.10% v/w) with fifty-eight constituents. The percentage of volatile oil produced by leaf (V.O.L) and callus (V.O.C) was quantitatively assessed over three independent experimental replicates. Statistical analysis was performed using an unpaired t-test to determine significant differences between the two groups. The mean yield from callus volatile oil (2.07 ± 0.12%) was significantly higher than that from leaf volatile oil (1.74 ± 0.10%), with a P-value of 0.0220 (t = 3.637, df = 4), indicating a statistically significant difference (*P* < 0.05). These results suggest that the callus possesses greater potential for volatile oil production than leaf under the conditions tested. Based on the GC-MS analysis, the chemical composition of *E. camaldulensis* leaf and callus oils were found to be closely related with remarkable qualitative and quantitative differences (Tables 3 and 4).

**Table 3:** Comparison between relative percentage of oil constituents between leaf and callus with high content in callus (*p* < 0.01).

Compound	Relative percent in VOL (% mean ± SD)	Relative percent in VOC (% mean ± SD)
1,8-cineole	20.54% ± 1.21	42.27% ± 2.45
α-pinene	8.81% ± 0.47	7.38% ± 0.39
α-terpineol	1.11% ± 0.12	4.22% ± 0.28
sabinene	0.84% ± 0.15	4.22% ± 0.31
cryptone	2.88% ± 0.19	1.64% ± 0.14
terpinen-4-ol	2.78% ± 0.22	1.54% ± 0.17
spathulenol	2.89% ± 0.24	1.51% ± 0.18
(-)-globulol	1.35% ± 0.16	0.73% ± 0.11
β- myrcene	1.01% ± 0.13	0.39% ± 0.21
(+)-aromadendrene	1.23% ± 0.26	0.13% ± 0.33

**Table 4:** Chemical composition of essential oil (EO) of callus volatile oil analyzed by GC-Mass spectrometry.

#	Compound	Area	%Area	Calculated LRI	LRI
1	α-Pinene	230,426,933	7.38%	929	933
2	Sabinene	131,752,870	4.22%	1184	1188
3	β-Myrcene	12,277,779	0.39%	993	990
4	α-Phellandrene	5,538,322	0.18%	1001	1005
5	1,8-Cineole	1,320,406,811	42.27%	1026	1030
6	γ-Terpinene	3,602,402	0.12%	1635	1631
7	4-Thujanol	1,133,800	0.04%	1092	1096
8	*p-*Cymenene	10,762,446	0.34%	1084	1090
9	Linalool	19,457,326	0.62%	1105	1100
10	Isoamylisovalerate	3,472,286	0.11%	1101	1104
11	cis-*p-*Menth-2-en-1-ol	4,957,366	0.16%	1124	1122
12	Neo-alloocimene	15,066,273	0.48%	1135	1131
13	Terpinene 1-ol	13,807,189	0.44%	1138	1140
14	(-)-trans-Pinocarveol	7,927,367	0.25%	1591	1591
15	β- Terpineol	21,785,551	0.70%	1153	1153
16	2,4-Nonadienal	4,526,751	0.14%	1567	1564
17	4-Isopropylcyclohexanone	7,068,208	0.23%	1572	1571
18	(+)-trans-Isolimonene	8,777,257	0.28%	981	983
19	4-Terpineol	48,184,249	1.54%	1177	1175
20	Cryptone	51,320,790	1.64%	1187	1184
21	α.-Terpineol	131,860,600	4.22%	1184	1188
22	γ.-Terpineol	18,770,799	0.60%	1195	1199
23	α-Phellandrene epoxide	5,051,974	0.16%	1183	1190
24	Verbenone	4,177,793	0.13%	1437	1441
25	trans-(+)-Carveol	7,520,086	0.24%	1222	1225
26	2-Hydroxy-1,8-cineole	5,165,098	0.17%	1477	1481
27	Cuminic aldehyde	16,851,176	0.54%	1234	1238
28	Δ-Carvone	7,018,454	0.22%	1240	1242
29	Thymoquinone	4,085,818	0.13%	1438	1441
30	Piperitone	6,215,858	0.20%	1250	1253
31	Phellandral	17,912,954	0.57%	1270	1273
32	1,3-*p-*Menthadien-7-al	3,191,077	0.10%	1280	1283
33	*p-*Cymen-7-ol	11,030,158	0.35%	1291	1295
34	Carvacrol	10,664,314	0.34%	1087	1090
35	trans-Sobrerol	3,685,585	0.12%	1628	1631
36	Β-Elemene	2,004,413	0.06%	1401	1405
37	α-Gurjunene	2,432,364	0.08%	1403	1409
38	Aromandendrene	20,021,255	0.64%	1436	1441
39	Alloaromadendrene	14,397,735	0.47%	1458	1458
40	Ledene	5,324,228	0.17%	1484	1481
41	Dehydroaromadendrene	1,699,050	0.05%	1591	1594
42	Alloaromadendrene oxide	1,830,282	0.06%	1401	1405
43	Epiglobulol	4,383,269	0.14%	1562	1564
44	1-Aromadendrene	4,064,768	0.13%	1444	1441
45	Spathulenol	47,051,378	1.51%	1571	1575
46	Globulol	22,937,802	0.73%	1576	1580
47	Viridiflorol	7,653,588	0.25%	1588	1591
48	Ledol	4,440,626	0.14%	1561	1564
49	Allospathulenol	2,053,902	0.07%	1575	1578
50	Isospathulenol	3,847,120	0.12%	1626	1631
51	t-Muurolol	4,646,903	0.15%	1643	1647
52	Isoaromadendrene epoxide	1,710,129	0.05%	1591	1594

Regarding quantitative differences, 1,8-Cineole, regarded as the main chemical component in the essential oils of *E. camaldulensis*, was found to be approximately 2.1-fold higher in VOC than in VOL. The amount of 1,8-cineole was found to be 20. 54% ± 1.21 in the leaf samples, while in the callus samples was quantified as 42.27% ± 2.45. To test the statistical significance, an unpaired t-test with Welch’s correction was used. The results showed a significant difference between the two groups (*p* = 0.0010, t = 13.62, df = 2.923). The difference is also supported by a 95% confidence interval from 16.39% to 26.59%, and the effect size (R²) was 0.9845, which shows a strong difference. These findings validate the claim that callus volatile oil contains 51.41% more than the amount of 1,8-cineole compared to leaf volatile oil (42.27% vs. 20.54%), and this difference is statistically significant (*p* < 0.01).

**Fig. 2:**
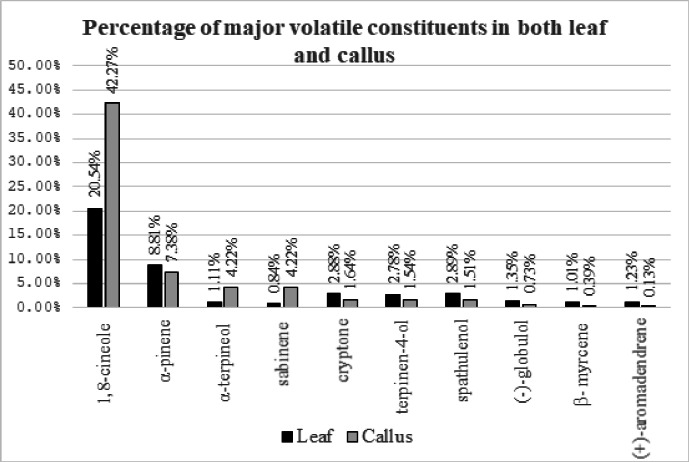
Comparison between percentage of major volatile constituents in both leaf and callus.

Similarly, α-terpineol and sabinene were found to be increased by more than 4 folds in VOC compared to VOL. Comparison between relative percentage of Volatile oil constituents between leaf and callus with high content in callus demonstrated in (Table 5). While multiple constituents were slightly varied between the callus and leaf volatile oil extracts as α-pinene, spathulenol, cryptone, terpinen-4-ol, (-)-globulol, (+)-aromadendrene, α-terpineol, β- myrcene and sabinene. Comparison between percentage of constituents’ classes in both leaf and callus is shown in (Fig. 2).

**Table 5:** Chemical composition of essential oil (EO) of leaf volatile oil analyzed by GC-Mass spectrometry.

#	Compound	Area	%Area	Calculated RI	LRI
1	α-Thujene	43,136,325	1.48%	925	929
2	α-Pinene	255,866,715	8.81%	936	933
3	Sabinene	24,465,284	0.84%	972	974
4	β-Pinene	3,597,849	0.12%	977	978
5	β-Myrcene	29,459,179	1.01%	989	990
6	α-Phellandrene	20,595,511	0.71%	999	1005
7	m-Cymene	378,835,020	13.05%	1012	1014
8	*p-*Mentha-1,5,8-triene	106,889,180	3.68%	1109	1111
9	1,8-Cineole	596,053,405	20.54%	1033	1030
10	γ.-Terpinene	2,846,405	0.10%	1166	1168
11	trans-Linalool oxide	1,960,608	0.07%	1433	1435
12	*p-*Cymenene	12,476,521	0.43%	1131	1131
13	Linalool	22,881,793	0.79%	1102	1100
14	Isoamyl isovalerate	5,064,071	0.17%	1285	1283
15	Thuj-4(10)-en-3-ol	2,262,318	0.08%	1214	1214
16	α-Thujone	1,497,869	0.05%	1913	1913
17	cis-*p*−2-Menthen-1-ol	5,793,899	0.20%	1127	1122
18	Neo-alloocimene	12,534,317	0.43%	1129	1131
19	1-Terpineol	7,843,252	0.27%	1141	1140
20	3,7-Octadiene-2-one	3,464,280	0.12%	1079	1077
21	3-Butyl-1-cyclohexene	7,913,137	0.27%	1137	1140
22	4-Isopropyl-cyclohexanone	9,357,904	0.32%	1569	1571
23	1-Methylnorcarane	3,803,202	0.13%	801	803
24	2,5-Dimethyl-2,4-hexadiene	4,858,520	0.17%	1281	1283
25	Terpinen-4-ol	80,716,030	2.78%	1173	1175
26	Cryptone	83,700,853	2.88%	1184	1184
27	α-Terpineol	32,097,629	1.11%	1188	1188
28	α-Phellandrene epoxide	12,207,153	0.42%	1191	1190
29	Piperitol	2,394,080	0.08%	1213	1214
30	3-Thujen-2-one	3,005,802	0.10%	1169	1168
31	4-Fluorobenzyl alcohol	1,869,606	0.06%	908	908
32	m-Cumenol	3,906,230	0.13%	1216	1221
33	2-Methyl-3-phenyl-propanal	30,389,663	1.05%	1241	1244
34	Piperitone	8,142,137	0.28%	1562	1564
35	Phellandral	26,531,607	0.91%	1271	1273
36	1,3-*p-*Menthadien-7-al	4,908,763	0.17%	1287	1283
37	*p-*Cymen-7-ol	14,020,964	0.48%	1299	1295
38	Carvacrol	9,507,021	0.33%	1295	1299
39	β-Elemene	4,142,345	0.14%	1400	1405
40	α-Gurjunene	6,105,768	0.21%	1408	1409
41	γ-Maaliene	1,955,666	0.07%	1434	1435
42	Calarene	1,598,749	0.06%	1469	1472
43	(+)-Aromadendrene	35,863,093	1.23%	1440	1441
44	9,10-Dehydroisolongifolene	1,346,601	0.05%	1911	1913
45	Alloaromadendrene	16,394,618	0.56%	1457	1458
46	Germacrene B	1,664,328	0.06%	1477	1480
47	Ledene	14,597,323	0.50%	1481	1481
48	γ-Cadinene	1,289,150	0.04%	1517	1517
49	δ-Cadinene	3,414,234	0.12%	1515	1511
50	Isoaromadendrene epoxide	1,088,661	0.04%	1514	1517
51	Epiglobulol	7,710,830	0.27%	1136	1140
52	Spathulenol	83,872,819	2.89%	1570	1575
53	(-)-Globulol	39,330,128	1.35%	1575	1580
54	Viridiflorol	13,527,645	0.47%	1585	1591
55	Ledol	8,180,216	0.28%	1561	1564
56	Allospathulenol	3,715,322	0.13%	1565	1570
57	Isospathulenol	9,086,205	0.31%	1628	1631
58	α-Cadinol	3,399,064	0.12%	1650	1653

In addition, the relative quantities of the rest of major peaks in VOL and VOC of *E. camaldulensis* varied significantly. For example, the relative percentages of α-pinene, spathulenol, cryptone, terpinen-4-ol, (-)-globulol, (+)-aromadendrene, α-terpineol, β- myrcene and sabinene in VOL were 8.81%, 2.89%, 2.88%, 2.78%, 1.35%, 1.23%, 1.11%, 1.01% and 0.84%, respectively. While their relative percentages in VOC were 7.38%, 1.51%, 1.64%, 1.54%, 0.73%, 0.13%, 4.22%, 0.39%, 4.22%, respectively as shown in (Table 5).

### Biological activities evaluation

#### Anti-inflammatory activity: Inhibition of TNF-α and IL-6 release in LPS-stimulated cells

Lipopolysaccharide (LPS), a key structural component of the outer membrane in gram-negative bacteria, is well-documented for its potent immunostimulatory effects. It is known to trigger systemic inflammatory responses, notably through the upregulation of pro-inflammatory cytokines such as tumor necrosis factor-alpha (TNF-α) and interleukin-6 (IL-6). TNF-α plays a central role in initiating the inflammatory cascade by promoting cytokine secretion, activating adhesion molecules, and stimulating immune cell proliferation. As an early responder to tissue damage, TNF-α serves as a critical regulatory point in many inflammatory conditions. Similarly, IL-6 exerts a wide range of biological activities and is considered a key mediator in the acute phase response to infection, injury, and inflammation. It enhances the production of acute-phase proteins like C-reactive protein (CRP), serum amyloid A, and fibrinogen. Additionally, IL-6 promotes the expression of adhesion molecules and angiogenesis, contributing to increased vascular permeability and the development of inflammatory edema.

**Fig. 3:**
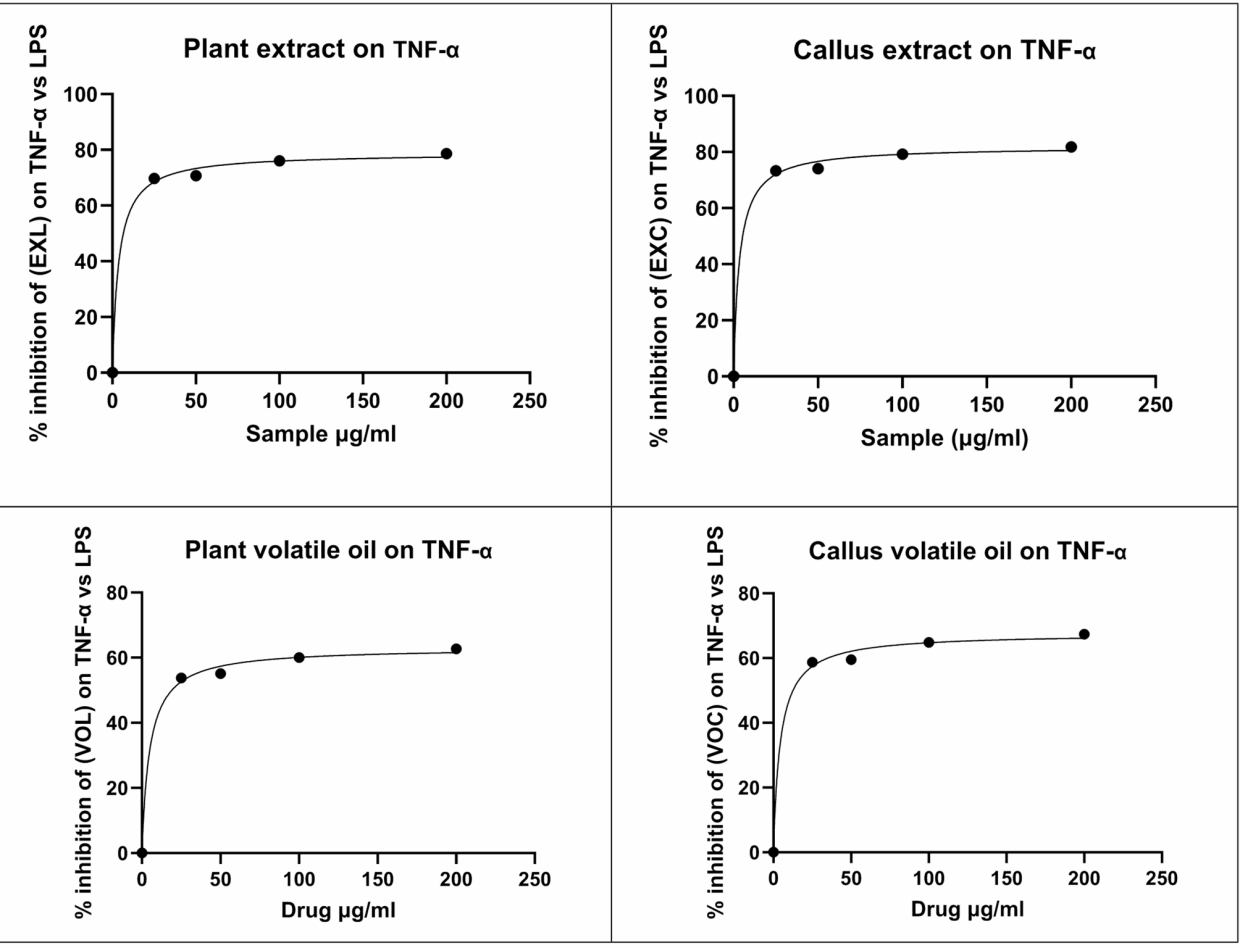
Dose response curve for serial concentration of *E. camaldulensis* extracts and percent inhibition of lipopolysaccharides on TNF- α.

**Fig. 4:**
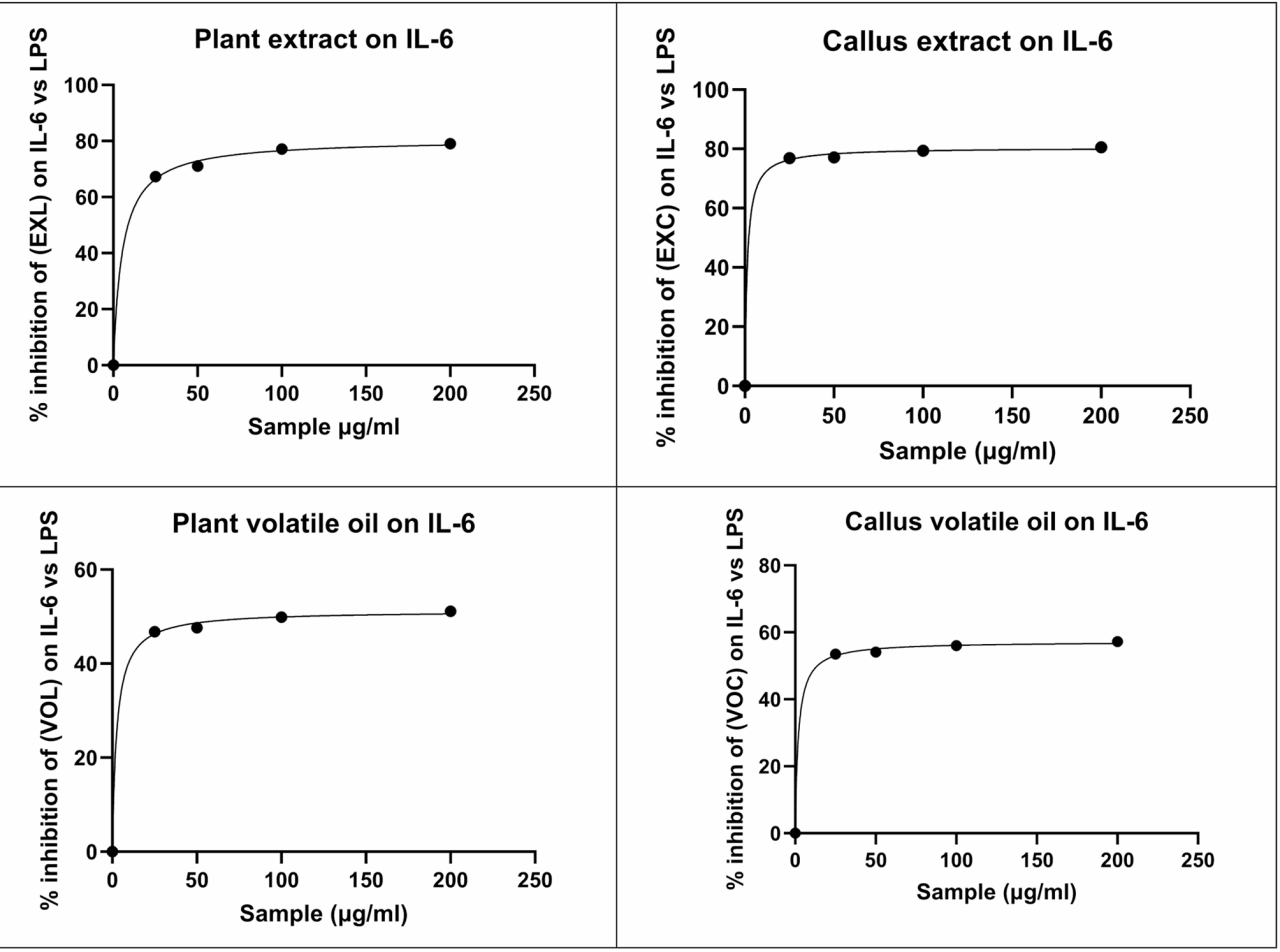
Dose response curve for serial concentration of *E. camaldulensis* extracts and percent inhibition of lipopolysaccharides on IL-6.

In this study, the ability of the tested extracts to suppress LPS-induced TNF-α and IL-6 production was evaluated in WI-38 fibroblast cells, following the methodology described by Rengasamy et al.^88^, using cells harvested from comparable growth plates. All examined extracts showed varying amounts of inhibitory activity and percentage of inhibition against the LPS-induced expression of TNF-α and IL-6 (Table 6) and (Table 7) with dose response curves (Fig. 3) and (Fig. 4).

**Table 6:** Concentration of produced TNF-α (Pg/ml) and percent Inhibition of lipopolysaccharides in tested extracts.

Extract (µg/ml)	E. camaldulensis methanolic extract of leaves (EXL) on TNF-α (pg/ml)	% inhibition of (EXL) on TNF-α vs. LPS	E. camaldulensis methanolic extract of callus (EXC) on TNF- α (pg/ml)	% inhibition of (EXC) on TNF-α vs. LPS	E. camaldulensis volatile oil of leaves (VOL) on TNF- α (pg/ml)	% inhibition of (VOL) on TNF-α vs. LPS	E. camaldulensis volatile oil of callus (VOC) on TNF- α (pg/ml)	% inhibition of (VOC) on TNF-α vs. LPS
0	300	0%	300	0%	300	0%	300	0%
25	90.8	69.73%	80.2	73.27%	138.7	53.77%	124	58.67%
50	88	70.67%	78	74%	134.7	55.1%	121.4	59.53%
100	71.9	76.03%	62.3	79.23%	120	60%	105.6	64.8%
200	64.1	78.63%	54.5	81.83%	111.8	62.73%	97.9	67.37%
Piroxicam (10 µg/mL)	65.4	78.2%	65.4	78.2%	65.4	78.2%	65.4	78.2%
IC50	3.783 µg/ml		3.439 µg/ml		4.981 µg/ml		4.448 µg/ml	

**Table 7:** Concentration of produced IL-6 (Pg/ml) and percent Inhibition of lipopolysaccharides in tested extracts.

Extract (µg/ml)	E. camaldulensis methanolic extract of leaves (EXL) on IL-6 (pg/ml)	% inhibition of (EXL) on IL-6 vs. LPS	E. camaldulensis methanolic extract of callus (EXC) on IL-6 (pg/ml)	% inhibition of (EXC) on IL-6 vs. LPS	E. camaldulensis volatile oil of leaves (VOL) on IL-6 (pg/ml)	% inhibition of (VOL) on IL-6 vs. LPS	E. camaldulensis volatile oil of callus (VOC) on IL-6 (pg/ml)	% inhibition of (VOC) on IL-6 vs. LPS
0	198.6	0%	198.6	0%	198.6	0%	198.6	0%
25	65	67.27%	45.9	76.89%	105.7	46.78%	92.4	53.47%
50	57.5	71.05%	45.5	77.09%	104	47.63%	91.2	54.08%
100	45.5	77.09%	40.9	79.41%	99.5	49.90%	87.3	56.04%
200	41.7	79.00%	38.7	80.51%	97.1	51.11%	84.9	57.25%
Piroxicam (10 µg/mL)	52.1	73.77%	52.1	73.77%	52.1	73.77%	52.1	73.77%
IC50	5.347 µg/ml		1.333 µg/ml		2.669 µg/ml		1.989 µg/ml	

In this study, the anti-inflammatory activity of the *Eucalyptus camaldulensis* extracts were evaluated by measuring their ability to inhibit LPS-induced release of the pro-inflammatory cytokines TNF-α and IL-6 in WI-38 fibroblast cells. In the LPS-stimulated group, TNF-α and IL-6 levels increased markedly to 300 and 198.6 pg/mL, respectively, confirming successful induction of an inflammatory response. Treatment with the reference anti-inflammatory drug piroxicam (10 µg/mL) significantly reduced these cytokines to 65.4 pg/mL for TNF-α and 52.1 pg/mL for IL-6, whereas the negative control showed basal cytokine levels of 51.2 pg/mL TNF-α and 39.1 pg/mL IL-6. All examined E. camaldulensis extracts (methanolic extracts of leaves and callus, EXL and EXC, and volatile oils of leaves and callus, VOL and VOC) produced a clear, dose-dependent inhibition of cytokine release over the concentration range 25–200 µg/mL. When expressed as percentage inhibition versus LPS, EXL inhibited TNF-α by approximately 69.7–78.6%, whereas EXC showed slightly higher inhibition (73.3–81.8%) across the same concentration range. VOL and VOC were less active on TNF-α, producing about 53.8–62.7% and 58.7–67.4% inhibition, respectively.

At the level of absolute cytokine concentrations, both methanolic extracts produced marked suppression of TNF-α and IL-6. For TNF-α, EXL yielded 90.8, 88.0, 71.9 and 64.1 pg/mL at 25, 50, 100 and 200 µg/mL, respectively, whereas EXC produced 80.2, 78.0, 62.3 and 54.5 pg/mL at the same doses. For IL-6, EXL gave 60.5, 57.5, 45.5 and 41.7 pg/mL, while EXC achieved 45.9, 45.5, 40.9 and 38.7 pg/mL at 25–200 µg/mL. Notably, at 100–200 µg/mL, both extracts reduced TNF-α to values approaching or slightly below those obtained with piroxicam (65.4 pg/mL) and close to the negative control (51.2 pg/mL), while IL-6 levels at the highest concentrations fell below those observed with piroxicam (52.1 pg/mL). EXC almost normalized IL-6, with values of 40.9–38.7 pg/mL that were essentially comparable to the basal level (39.1 pg/mL). These findings highlight a robust anti-inflammatory effect of both methanolic extracts, with EXC consistently outperforming EXL at matched concentrations.

The volatile oils also exhibited dose-dependent inhibition of TNF-α and IL-6, although to a lesser extent than the methanolic extracts. For TNF-α, treatment with VOL reduced the cytokine to 138.7, 134.7, 120.0 and 111.8 pg/mL at 25, 50, 100 and 200 µg/mL, respectively, whereas VOC yielded lower corresponding values of 124.0, 121.4, 105.6 and 97.9 pg/mL. For IL-6, VOL gave 105.7, 104.0, 99.5 and 97.1 pg/mL, while VOC produced 92.4, 91.2, 87.3 and 84.9 pg/mL over the same dose range. These data confirm that the callus oil (VOC) consistently outperformed the leaf oil (VOL) for both cytokines. However, although both oils were clearly active compared with the LPS condition, neither matched the maximal cytokine suppression achieved by piroxicam nor reached the basal levels attained by the highest doses of the methanolic extracts.

The concentration–response data were further analyzed by non-linear regression to estimate IC₅₀ values (Tables 6 and 7). For TNF-α, EXC exhibited the lowest IC₅₀ (3.439 µg/mL), followed by EXL (3.783 µg/mL), VOC (4.448 µg/mL) and VOL (4.981 µg/mL). For IL-6, EXC again showed the greatest potency (IC₅₀ = 1.333 µg/mL), followed by VOC (1.989 µg/mL) and VOL (2.669 µg/mL), whereas EXL displayed the highest IC₅₀ (5.347 µg/mL). The lower IC₅₀ values of EXC for both cytokines indicate that smaller concentrations are required to achieve 50% inhibition, confirming the superior anti-inflammatory potency of the callus methanolic extract compared with the other extracts. VOC also demonstrated better potency than VOL, in line with its stronger inhibition of TNF-α and IL-6 at matched doses. Taking together, the combined TNF-α and IL-6 data, expressed both as absolute cytokine levels and as percent inhibition versus LPS, indicate that all *E. camaldulensis* extracts possess marked anti-inflammatory activity, with a clear rank order of potency. Among the methanolic extracts, EXC is consistently more effective than EXL, which may be attributed to its relatively higher levels of flavonoids and phenolic acids and/or improved bioavailability of these constituents in the callus matrix^89^. Among the volatile oils, VOC is more active than VOL, a trend that may be related to its elevated content of 1,8-cineole, α-terpineol and sabinene, compounds previously reported to exert anti-inflammatory effects^90^. When compared with the reference NSAID piroxicam, the plant extracts showed comparable inhibitory effects at their higher tested concentrations, especially EXC and, to a lesser extent, EXL. EXC at 200 µg/mL produced TNF-α and IL-6 inhibitions (81.83% and 80.51%, respectively) that were slightly higher than those of piroxicam at 10 µg/mL (78.2% and 73.77%, respectively), while EXL approached the standard, particularly for IL-6. The ability of EXC to achieve equal or greater suppression of both TNF-α and IL-6 than piroxicam strongly supports its potential as a promising anti-inflammatory candidate. Across all treatments and concentrations, TNF-α and IL-6 inhibition were strongly positively correlated (*r* = 0.96, *p* < 0.0001), indicating that samples that effectively suppressed TNF-α also tended to strongly inhibit IL-6. A strong positive correlation was also observed between 1,8-cineole content and IL-6 inhibition (*r* = 0.89, *p* = 0.003), indicating that the oil richer in 1,8-cineole (VOC) generally produced greater cytokine suppression than VOL.

Overall, these findings demonstrate that *E. camaldulensis* callus, particularly its methanolic extract (EXC), possesses the highest anti-inflammatory potential among the tested preparations. The superior activity of EXC and VOC underscores the value of callus culture as a biotechnological tool to enhance or modulate the phytochemical profile and biological activity of *E. camaldulensis*. Future studies should focus on elucidating their molecular mechanisms of action and validating these in vitro observations in appropriate in vivo models.

#### Antioxidant activity

Percent reduction of absorbance for *E. camaldulensis* methanolic extract (EXC) (88.48%) and volatile oil (VOC) (82.1%) of callus is higher by nearly 25% than methanolic extract (EXL) (70.78%) and volatile oil (VOL) (64.81%) of *E. camaldulensis* leaf, respectively. The obtained results indicate intermediate to potent antioxidant capacity that may be correlated to the anti-inflammatory activity of callus volatile oil and extract. The detected antioxidant activity of the studied extracts can be explained by the quantities of 1,8-cineole, potent antioxidant, callus volatile oil containing a higher proportion of 1,8-cineole than leaf volatile oil (42.27% vs. 20.54%). Beside the high concentrations of flavonoids, with the known antioxidant activity, in callus extracts compared to leaf extract^91^. The percentage scavenging of DPPH radical, expressed as Trolox equivalent antioxidant capacity (TEAC), was found to be about 9.01 µg/ml and 11.63 µg/ml for the volatile oil of *E. camaldulensis* leaf and callus, respectively. This agrees with previous reports that revealed the potent antioxidant potential of Eucalyptus oil^92^.

Percentage of absorbance reduction in the DPPH assay and TEAC of the tested extracts were demonstrated in (Table S.3 & Table S.4 & Figure S.3).

## Conclusion

The current study demonstrated the first comprehensive phytochemical investigation into the enhancement of secondary metabolite production of *Eucalyptus camaldulensis* using suspension culture technique. The results demonstrate that callus extracts contain significantly higher concentrations of key bioactive compounds, such as 1,8-cineole, α-terpineol, and sabinene, compared to leaf extracts. Bioassay analyses revealed that callus-derived volatile oil exhibited superior anti-inflammatory and antioxidant activities, with its anti-inflammatory effect surpassing that of piroxicam. These findings underscore the potential of in vitro tissue culture as an effective biotechnological approach to enhancing the yield and bioactivity of secondary metabolites in *E. camaldulensis*, paving the way for the development of more potent natural therapeutic agents.

Further in vivo investigations are warranted to validate the therapeutic efficacy and safety of the bioactive extracts. Complementary advanced statistical analyses are also recommended to identify and prioritize the lead compounds contributing to the observed biological activities. Future studies may focus on optimizing culture conditions through the strategic use of various plant growth regulators, aiming to improve both the yield and specificity of targeted phytochemicals. Additionally, mechanistic studies at the cellular and molecular levels are essential to elucidate the underlying pathways mediating the biological effects of these extracts.

## Supplementary Information

Below is the link to the electronic supplementary material.

Supplementary Material 1

## Data Availability

The datasets generated and/or analysed during the current study are not publicly available to prevent misuse when published but are available from the corresponding author on reasonable request.
