# Supplementary material for: Enhancing anti-inflammatory activity of Eucalyptus camaldulensis by upregulating secondary metabolites using suspension cultures techniques
Source: Sci Rep. 2026 Jan 29;16:4090. doi: 10.1038/s41598-025-34963-8 (PMC12855876; doi:10.1038/s41598-025-34963-8)
Supplement: Supplementary file 1 — Supplementary Material 1 [file 41598_2025_34963_MOESM1_ESM.docx]

**Supplementary Data**

**Enhancing anti-inflammatory activity of *Eucalyptus camaldulensis* by upregulating secondary metabolites using suspension cultures techniques**

Mahrous H. Mahrous*^a^, Atef MK Nassar^b^, Fathy K. EL-Fiky^a^, Hala M. Hammoda^c^, Amr El-Hawiet^c^

^a^ Department of Pharmacognosy, Faculty of Pharmacy, Delta University for Science and Technology, Dakhliya, Egypt.

^b^ Department of Plant Protection, Faculty of Agriculture, Damanhour University, Damanhour, Egypt

^c^ Department of Pharmacognosy, Faculty of Pharmacy, Alexandria University, Alexandria 21521, Egypt

Corresponding author email address: [mahrous.hisham@deltauniv.edu.eg](mailto:mahrous.hisham@deltauniv.edu.eg)
[mahrous.hesham91@yahoo.com](mailto:mahrous.hesham91@yahoo.com)


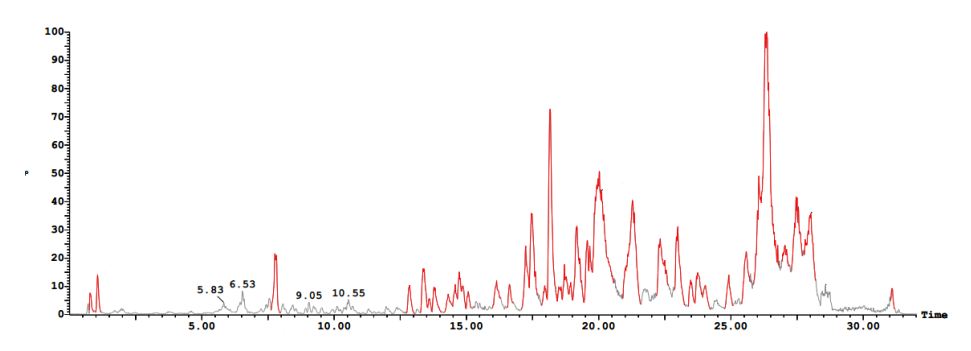


Figure S.1: UPLC chromatogram of *Eucalyptus camaldulensis* leaf methanolic extract.


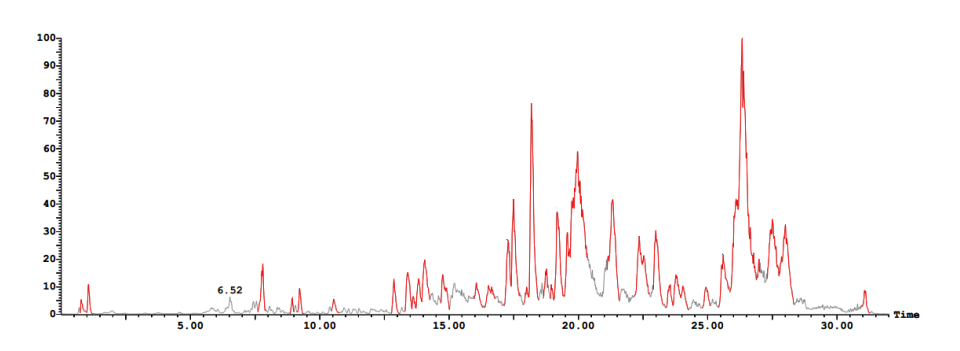


Figure S.2: UPLC chromatogram of *Eucalyptus camaldulensis* callus methanolic extract

Table S.1: Linearity and sensitivity parameters for Kaempferol-4′- glucoside, Caffeic acid, Gallic acid, and Maslinic acid

| **Compound** | **Linearity range (mg/ml)** | **Slope (a)** | **Intercept (b)** | **Correlation coefficient (r)** | **LOD (mg/ml)** | **LOQ (mg/ml)** |
| --- | --- | --- | --- | --- | --- | --- |
| **Kaempferol-4′-** **glucoside** | 0.02-0.30 | 8.42*10^6^ | -1.21*10^5^ | 0.997 | 0.008 | 0.025 |
| **Caffeic acid** | 0.05-0.51 | 2.32*10^7^ | -8.02*10^4^ | 0.994 | 0.013 | 0.043 |
| **Gallic acid** | 0.03-0.43 | 4.15*10^7^ | -7.21*10^4^ | 0.994 | 0.009 | 0.029 |
| **Maslinic acid** | 0.04-0.61 | 1.52*10^7^ | -9.13*10^4^ | 0.996 | 0.014 | 0.046 |

Experimental conditions as in section 2.3.3 for each calibration curve the equation is y=ax + b, where y is the peak area, x is the concentration of the standard (mg/ml), a is the slope, b is the intercept, r the correlation coefficient, LOD is the limit of detection and LOQ is the limit of quantitation.

Table S.2: Comparison between relative percentage of oil constituents between leaf and callus with high content in callus

| Compound | Relative percent in VOL | Relative percent in VOC |
| --- | --- | --- |
| 1,8-cineole | 20.54% | 42.27% |
| α-pinene | 8.81% | 7.38% |
| α-terpineol | 1.11% | 4.22% |
| sabinene | 0.84% | 4.22% |
| cryptone | 2.88% | 1.64% |
| terpinen-4-ol | 2.78% | 1.54% |
| spathulenol | 2.89% | 1.51% |
| (-)-globulol | 1.35% | 0.73% |
| β- myrcene | 1.01% | 0.39% |
| (+)-aromadendrene | 1.23% | 0.13% |

Table S.3: Absorbance and percentage inhibition of different concentrations of Trolox (12.5-0.3) µg/ml in the DPPH assay.

| Trolox concentration (µg/ml) | Absorbance | % inhibition |
| --- | --- | --- |
| 12.5 | 0.046 | 85.98984772 |
| 6.25 | 0.162 | 50.65989848 |
| 3.125 | 0.246 | 25.07614213 |
| 0.78125 | 0.286 | 12.89340102 |
| 0.390625 | 0.313 | 4.670050761 |

Figure S.3: Percent of inhibition in DPPH assay and TEAC

Table S.4: Percentage of absorbance reduction in the DPPH assay and TEAC:

| Sample | Sample absorbance | % Inhibition of DPPH | TEAC |
| --- | --- | --- | --- |
| *Eucalyptus camaldulensis* methanolic extract of callus (EXC) | 0.08 | 88.48% | 12.6072 μg/ml |
| *Eucalyptus camaldulensis* volatile oil of callus (VOC) | 0.13 | 82.1% | 11.63769 μg/ml |
| *Eucalyptus camaldulensis* methanolic extract of leaves (EXL) | 0.2 | 70.78% | 9.917588 μg/ml |
| *Eucalyptus camaldulensis* volatile oil of leaves (VOL) | 0.25 | 64.81% | 9.010627 μg/ml |
